# Modifying Bodily Self-Awareness during Acupuncture Needle Stimulation Using the Rubber Hand Illusion

**DOI:** 10.1155/2013/849602

**Published:** 2013-04-03

**Authors:** Dong-Seon Chang, Yun-Ji Kim, Soon-Ho Lee, Hyejung Lee, In-Seon Lee, Hi-Joon Park, Christian Wallraven, Younbyoung Chae

**Affiliations:** ^1^Acupuncture and Meridian Science Research Center, Kyung Hee University, 1 Hoegi-dong, Dongdaemun-gu, Seoul 130-701, Republic of Korea; ^2^Department of Human Perception, Cognition and Action, Max Planck Institute for Biological Cybernetics, Tübingen, Germany; ^3^Department of Brain and Cognitive Engineering, Korea University, Seoul, Republic of Korea

## Abstract

*Background*. The rubber hand illusion (RHI) is an experimental paradigm that manipulates important aspects of body self-awareness. *Objectives*. We were interested in whether modifying bodily self-awareness by manipulation of body ownership and visual expectations using the RHI would change the subjective perception of pain as well as the autonomic response to acupuncture needle stimulation. *Methods*. Acupuncture needle stimulation was applied to the real hand during the RHI with (experiment 1) or without (experiment 2) visual expectation while measuring concurrent autonomic changes such as the skin conductance response (SCR). Subjective responses such as perception of the RHI and perceived pain were measured by questionnaires. *Results*. In experiment 1, the amplitude of the increase in SCR was visibly higher during the synchronous session compared with that of the asynchronous session. In experiment 2, the amplitude of the increase of SCR was lower for the synchronous session compared with that for the asynchronous session. Comparing these two experiments, the visual expectation of needle stimulation produced a greater autonomic response to acupuncture stimulation. *Conclusions*. Our findings suggest that the sympathetic response to acupuncture needle stimulation is primarily influenced by visual expectation rather than by modifications of body ownership.

## 1. Introduction

What is the role of body self-awareness or self-consciousness in a clinical context? Imagine yourself receiving acupuncture needle treatment. Would you expect a difference in your perception depending on whether you looked at the site of needle penetration? Next, imagine a patient with asomatognosia or somatoparaphrenia (a patient who cannot feel his/her body or does not recognize parts of the body as his/her own) receiving moxibustion on a part of the body that is not perceived as self. Would you expect the treatment to have the same effects as with a normal patient who could feel all of the sensations of heat, touch, and pain on the skin? 

These questions clearly demonstrate the importance of body awareness or bodily self-consciousness in clinical treatments. Body awareness and self-consciousness are thought to include both an attentional focus (e.g., visual attention) and awareness of bodily sensations (e.g., proprioception and interoception) [1–4]. Although it is difficult to find unquestionably clear definitions, currently, aspects of bodily self-awareness or self-consciousness are typically investigated by modifying participants' senses of body ownership and embodiment [1, 5, 6]. Body ownership is defined as the sense that the body or body part belongs to oneself (i.e., self-attribution) [7, 8], whereas a sense of embodiment is defined as the experience of being within the borders of one's body (i.e., self-localization) [5, 6, 8, 9]. These senses can be experimentally modified using different perceptual illusions such as the out-of-body illusion, the body-swapping illusion, or most commonly, the rubber hand illusion (RHI) [10, 11].

The RHI is evoked when a subject watches a rubber hand being stroked or touched while their own hand is stroked or touched simultaneously, leading to the experience of additional body ownership such that the rubber hand also “feels like one's own hand” [12]. This illusion is thought to arise due to the multimodal integration of the senses of vision, somatosensation (e.g., touch), and proprioception (e.g., position) in the brain [13]. The visual stimulation of the rubber hand matching the tactile stimulation of the actual hand leads to a stable and strong experience of illusory body ownership (embodiment) of the rubber hand, accompanied by a sense of body disownership (disembodiment) of the actual hand. Evidence for illusory body ownership has been found in functional magnetic resonance imaging and skin conductance response (SCR) studies, where threatening the rubber hand during the illusion elicits a measurable cortical anxiety response, indicating that the brain has indeed accepted the rubber hand as part of its own body [14, 15]. Thus, the RHI has been repeatedly shown to be an effective experimental tool for manipulating the sense of body ownership and thereby modifying aspects of bodily self-awareness [16, 17].

Acupuncture, an ancient East Asian therapeutic technique, uses needles to penetrate the skin and needle manipulation to stimulate the body [18]. The specific perception of acupuncture treatment is traditionally termed the *deqi* sensation and includes a sense of soreness and aching that can be experienced as dull pain [19, 20]. Sympathetic responses following acupuncture needle stimulation indicate enhanced SCR in response to both real and sham acupuncture [21, 22]. The clinical effects of acupuncture treatment (e.g., analgesic effects) have been investigated and confirmed in a number of studies in animals and in clinical populations, and many researchers have contributed to identifying the brain processes activated by acupuncture [23]. Nevertheless, the underlying mechanisms of acupuncture and its mode of action are unclear, and psychosocial and contextual factors such as expectation, attention, and body schema may play important roles in the clinical effects of acupuncture [24]. However, no studies are available in which different aspects of bodily self-awareness have been actively manipulated and investigated. 

In the current study, we were interested in actively manipulating some aspects of bodily self-awareness through modification of body ownership and visual expectations using the RHI. How would physiological and subjective responses to acupuncture needle stimulation differ during embodied compared with disembodied body conditions? How would these responses differ based on visual feedback during acupuncture stimulation? The aim of our study was to investigate whether these modifications of bodily self-awareness result in different psychophysiological responses when participants are treated with acupuncture needle stimulation. 

## 2. Methods and Materials

### 2.1. Participants

Thirty-one participants (age, 19–29 years; 16 males and 15 females) recruited by advertisement from the general population of students, staff, and visitors to Kyung Hee University, Seoul, Republic of Korea, participated in the experiments. Nineteen participants (nine males and 10 females) took part in experiment  1, and 12 participants (seven males and five females) took part in experiment  2. The participants received 10,000 Korean Won (approximately 10 USD) for reimbursement. All participants received a detailed explanation of the study, and written informed consent was obtained. This investigation was conducted in accordance with the guidelines of the human subjects committee of Kyung Hee University.

### 2.2. Experimental Design

**Figure 1:**
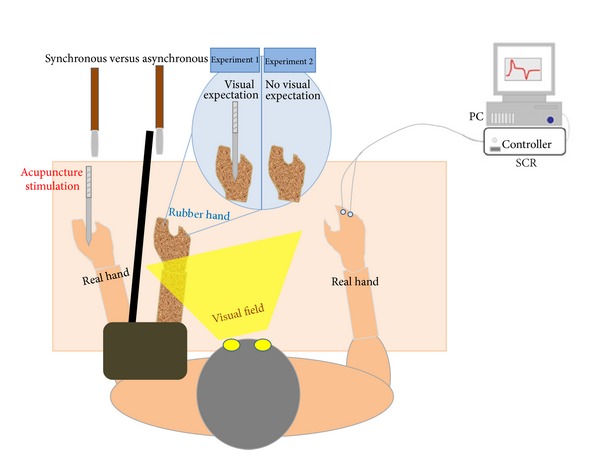
Schematic drawing of the experimental setup illustrating the rubber hand illusion with (experiment  1) and without (experiment  2) the visual expectation when participants received acupuncture stimulation on their real hand. Two small paintbrushes stroked the rubber hand and the participant's hidden real left hand as synchronously as possible under one condition (synchronous condition) and asynchronously under the other (asynchronous condition).

A rubber hand was placed in front of each participant while his/her left hand was hidden from sight. The setup followed standard procedure and was designed almost identically to a previous study [25] (Figure 1). Both experiments followed a within-participants repeated-measures design, and the independent variable was a synchronous versus an asynchronous brush touch on the hand. In both experiments, participants received acupuncture needle stimulation on the hidden (real) left hand immediately after commencing the RHI in the synchronous condition and the control trial in the asynchronous condition. Experiments  1 and 2 were designed almost identically except that in experiment  1, the participants saw the acupuncture needle stimulation applied to the rubber hand in a synchronized and colocalized manner as it was applied to the real hand (visual expectation condition), whereas in experiment  2, they received no visual feedback at all (no visual expectation), meaning that they did not know when or where the acupuncture needle would penetrate their real hand. Thus, a between-subjects design was additionally tested with visual cue as the independent variable (presence or absence of acupuncture needle penetration of the rubber hand at the time of acupuncture needle penetration of the real hand). 

### 2.3. Procedures

#### 2.3.1. Rubber Hand Illusion Induction

The participants were told to fixate on the rubber hand (Korean Prosthetic Limbs Research Institute, Seoul, Korea) and not to look elsewhere. They were also not allowed to move any of their fingers. A small tube was preinstalled on the rubber hand and the real hand in experiment  1, and the acupuncture needle stimulation was followed by tapping the inserted needle on the tube. Two small paintbrushes stroked the rubber hand and the participant's hidden real left hand as synchronously as possible under one condition (synchronous condition) and asynchronously under the other (asynchronous condition). After 300 seconds of brush stroking, an acupuncture needle was applied to the real hand by inserting the needle into the skin using the small tube. The same procedure was repeated twice, once with synchronous brush stroking and once with asynchronous brush stroking. The order was randomized, so participants were randomly assigned to one of two groups; that is, they received either the synchronous or the asynchronous session first. The participants had to wait 10–15 minutes between the two sessions. 

#### 2.3.2. Acupuncture Stimulation

Participants were first informed about the experiment and told that physiological data including skin conductance measurements would be taken while they received acupuncture treatment. The participants were told that they would randomly receive either real acupuncture treatment (with needle penetration of the skin) or sham acupuncture treatment (without needle penetration) for each trial to produce uncertainty as to whether they would receive real acupuncture needle stimulation. The Park sham needle was explained and demonstrated in front of all participants. All participants were told that they would participate twice in the same procedure. Then, the participants were led into the experimental room and seated with their left and right arms on a table. They all received acupuncture stimulation with the real needle at acupoint LI4, on the dorsum of the left hand, radial to the midpoint of the second metacarpal bone, in the left hand in front of a curtain. The needles (Dongbang Acupuncture, Inc., Gyeonggi-do, Korea) were 0.25 mm in diameter and 40 mm long. The needles for the demonstration of the sham procedure (Park sham needle; Dongbang Acupuncture) were identical in size and appearance.

#### 2.3.3. Skin Conductance Response Measurement

Two electrodes were placed on the left hand to measure skin conductance. Skin conductance was recorded from the medial phalanges of the second and third digits of the left hand, with 0.05 M NaCl paste as the electrolyte. Skin conductance was digitized and recorded with a galvanic skin response amplifier (GSR Amp ML116; ADInstruments, Bella Vista, Australia) and a high-performance data acquisition PowerLab 8/30 system (ML870; ADInstruments). Each half-second was deviated from a 1 s baseline prior to inserting the acupuncture needle (cue onset) and averaged across the 15 s of each acupuncture session to assess responses during and after acupuncture treatment, resulting in change scores that reflected increases or decreases from baseline. 

#### 2.3.4. Rubber Hand Illusion Questionnaire

After finishing each session (synchronous and asynchronous), the participants reported their perception of the RHI using the Rubber Hand Illusion Perception Scale, which includes nine questions [12]. The participants were also required to give detailed answers to an open-end questionnaire asking about their experience and changes in their perception during the experiment.

#### 2.3.5. Self-Reported Pain Rating

Subjective pain ratings were obtained immediately after each acupuncture stimulation session. The participants evaluated acupuncture-induced pain using a 100 mm visual analogue scale.

### 2.4. Data Analysis

All values are expressed as mean ± standard error. The RHI ratings during the synchronous brush stroking (induction of illusory body ownership) and the asynchronous brush stroking session (control) were compared using a paired *t-*test. The subjective pain ratings and the SCR responses were analyzed by mixed between-group analyses of variance (ANOVAs) with one (time) repeated-measure factor. The level of significance was set at 0.05 for all analyses. Statistical analyses were performed using the Statistical Package for Social Sciences for Windows 17.0 (SPSS, Inc., Chicago, IL, USA). 

## 3. Results

### 3.1. Self-Assessments of the Rubber Hand Illusion

**Figure 2:**
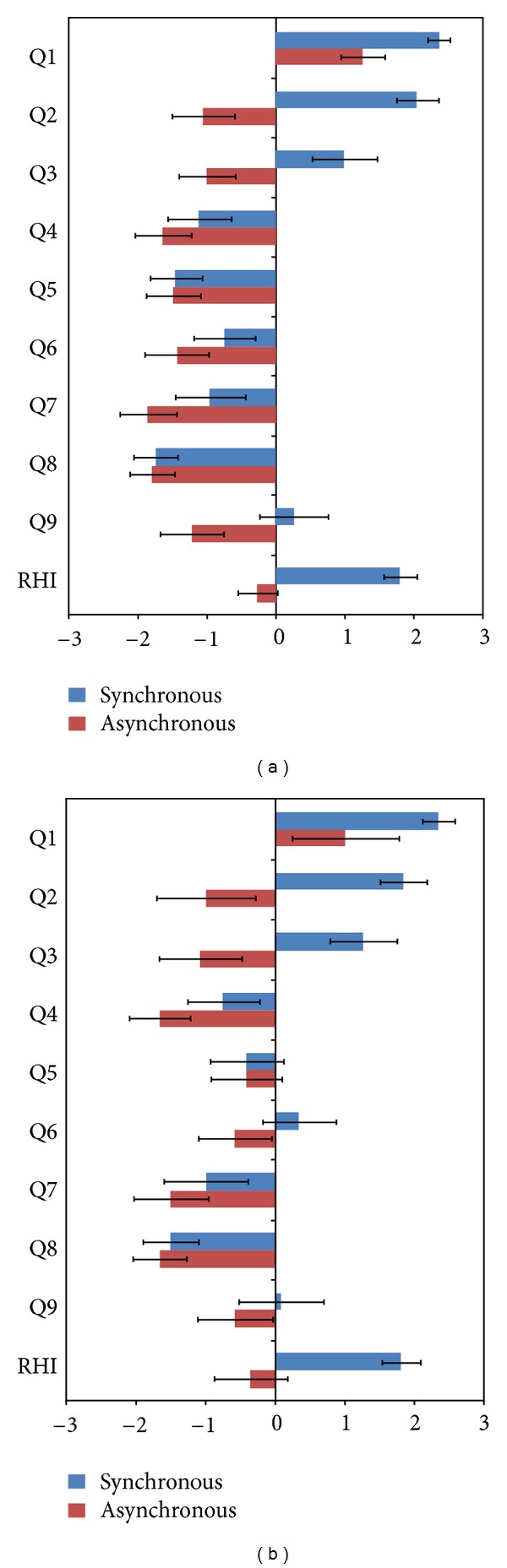
The Rubber Hand Illusion Perception Scale. Q1: It seemed as if I were feeling the touch of the paintbrush in the location where I saw the rubber hand touched. Q2: It seemed as though the touch I felt was caused by the paintbrush touching the rubber hand. Q3: I felt as if the rubber hand was my hand. Q4: I felt as if my (real) hand were drifting toward the right (toward the rubber hand). Q5: It seemed as if I had more than one left hand or arm. Q6: It seemed as if the touch I was feeling came from somewhere between my own hand and the rubber hand. Q7: It felt as if my (real) hand were turning “rubbery.” Q8: It appeared (visually) as if the rubber hand were drifting towards the left (towards my hand). Q9: The rubber hand began to resemble my own (real) hand, in terms of shape, skin tone, freckles, or some other visual feature. The first three questions (Q1–Q3) were designed to correspond to the rubber hand illusion. Mean responses to the rubber hand illusion questionnaire statements on a 7-point Likert scale ranging from “strongly disagree (−3)” to “strongly agree (+3),” with standard errors. A significant difference was observed between the synchronous and asynchronous brush stroking sessions under the visual expectation (1.8 ± 0.2 versus −0.3 ± 0.3, *t* = 5.883, *P* < 0.001, (a)) and no visual expectation conditions (1.8 ± 0.3 versus −0.4 ± 0.3, *t* = 5.933, *P* < 0.001, (b)). Values are mean ± standard error.

A significant difference was observed in the self-report RHI questionnaire between the synchronous and asynchronous brush stroking sessions in both experiment  1 (visual expectation condition) (1.8 ± 0.2 versus −0.3 ± 0.3, *t* = 5.883, *P* < 0.001, Figure 2(a)) and experiment  2 (no visual expectation condition) (1.8 ± 0.3 versus −0.4 ± 0.3, *t* = 5.933, *P* < 0.001, Figure 2(b)). 

### 3.2. Self-Assessments of Pain

No significant differences were observed between the synchronous and asynchronous brush stroking sessions in self-reported pain in experiment  1 (visual expectation condition) (4.1 ± 0.6  versus  3.5 ± 0.6) or experiment  2 (no visual expectation condition) (2.8 ± 0.6  versus  2.5 ± 0.6) (*F*_[3,46]_ = 1.215, *P* > 0.315).

### 3.3. Skin Conductance Response

**Figure 3:**
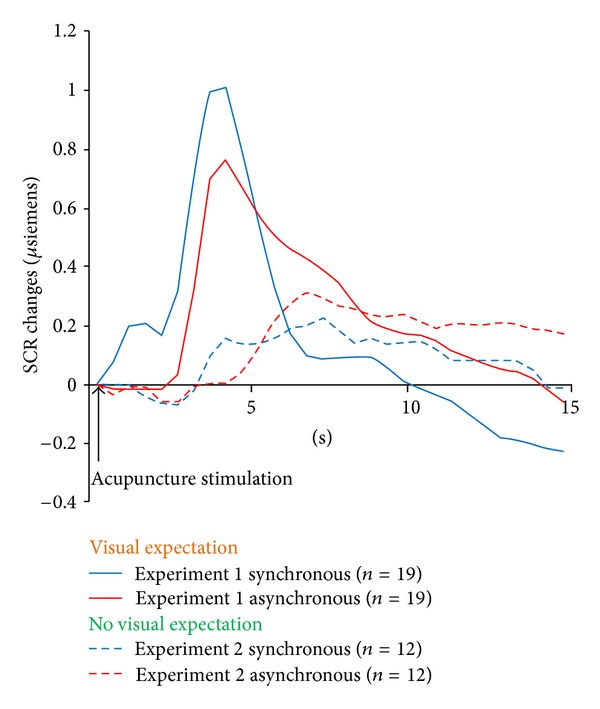
Skin conductance response (SCR) recordings are presented as the mean change in SCR over time. A significant condition ((synchronous or asynchronous session) × (with or without visual expectation condition)) × time effect was observed (*F*_[3,116]_ = 1.610, *P* < 0.001).

The SCR recordings are presented as the mean SCR change over time (Figure 3). A 4 × 30 repeated-measures ANOVA was conducted for each SCR recording during acupuncture stimulation, with condition ((synchronous or asynchronous session) × (with or without visual expectation condition)) as the between-subjects factor and time (measured every 15 s) as the within-subjects factor. The repeated-measures ANOVA showed a significant effect of time (*F*_[3,116]_ = 3.406, *P* < 0.001) and a condition × time interaction effect (*F*_[3,116]_ = 1.610, *P* < 0.001). 

## 4. Discussion

We modified different aspects of bodily self-awareness using the RHI and observed psychophysiological responses to acupuncture needle stimulation. Bodily self-awareness was manipulated by modifying body ownership (synchronous and asynchronous brush stroking sessions) and visual expectations (with and without visual cues in experiments  1 and 2, resp.). No significant differences in subjective pain ratings were found for the modification of body ownership, but the visual expectation of needle stimulation seemed to determine the patterns of autonomic responses. The disrupted sense of body ownership appeared to notably ameliorate the increase in SCR in response to acupuncture stimulation.

### 4.1. Bodily Self-Awareness during Rubber Hand Illusion

This is one of the first studies applying the RHI experimental paradigm (Figure 1) to modify aspects of bodily self-awareness to examining differential responses to acupuncture needle stimulation. Participants' answers to the RHI questionnaire confirmed the stable and successful evocation of the RHI in both experiment  1 (visual expectation condition) and experiment  2 (no visual expectation condition), as the significant differences between the synchronous and asynchronous brush stroking sessions showed (Figure 2). This result confirms that synchronous brushing of the visible rubber hand and of the participant's own hand successfully produced the sense of body ownership and embodiment for the rubber hand while inducing a disruption of body ownership and disembodiment for the real hand. These results are comparable to those of the initial study describing the RHI [12].

### 4.2. Pain Perception during Rubber Hand Illusion

Acupuncture needles were always applied to the real hand, implying a disruption of body ownership (disembodiment) during the synchronous sessions when the RHI was successfully evoked and a normal sense of body ownership (embodiment) during the asynchronous sessions. We wanted to observe differences in the responses to acupuncture stimulation based on the embodied and disembodied conditions. The question of what happens with the actual hand during the illusion has been a point of recent discussion [17, 26, 27]. Does inducing illusory body ownership result in disembodiment or actual disownership of the real hand? Recent evidence points toward a displacement of sensations from the actual hand, demonstrating that disembodiment induces cooling of the disembodied body part as well as desensitization to tactile stimuli [17, 27]. Furthermore, increased histamine reactivity has been reported during the RHI in the real arm, indicating a rejection of the “replaced” hand by the innate immune system, similar to autoimmune disorders [28]. Previous studies using other body illusions have also reported modulation of perceived pain when body ownership was manipulated [29, 30]. Therefore, we initially expected a difference in subjective pain perception in the disembodied hand during acupuncture stimulation. However, no significant differences were found in the subjective pain ratings across all sessions. Even more surprising was the finding that the subjective pain ratings did not significantly differ between experiments  1 and 2. Previous reports have emphasized the role of vision in bodily self-awareness and body ownership [31, 32]. For example, seeing one's own body while receiving nociceptive stimuli induces an analgesic effect, implicating the involvement of bodily self-awareness and body ownership because seeing another person's body has no effect [33]. Nonetheless, the results of a very recently published study are consistent with our results, as they also reported no differences in subjective pain perception during the RHI [26].

### 4.3. Autonomic Responses during Rubber Hand Illusion

Although subjective pain ratings did not differ, autonomic responses showed visible differences for the different conditions of modified bodily self-awareness. This result was also consistent with one of our previous studies in which subjective experiences and physiological responses to real and sham acupuncture stimulation differed from each other [21]. The autonomic responses depended primarily on visual expectation, but they were also influenced by body ownership (Figure 3). The increase in SCR to acupuncture stimulation was significantly higher under the visual expectation condition (experiment  1) compared with the no visual expectation condition (experiment  2). This could be due to the already reported role of vision in bodily self-awareness and body ownership [31–33]. When no vision of the acupuncture needle stimulation was provided (experiment  2), the amplitude of the increase of SCR was lower for the synchronous session compared with that for the asynchronous session (Figure 3), suggesting that sympathetic activation in response to acupuncture needle stimulation decreased in the disembodied condition. Consistent with previous studies where disruption of body ownership reduced temperature and tactile sensitivity in the disembodied hand [17], this could mean that the psychologically induced limb-specific disruption reduced the physiological autonomic response to acupuncture stimulation. When a visual expectation of the acupuncture needle stimulation existed (experiment  1), the amplitude of the increase in SCR was visibly higher during the synchronous session compared with that of the asynchronous session (Figure 3). In this case, the participants seemed to have already allowed the incorporation of the artificial body part into their “self-representation,” as indicated by the RHI questionnaire responses, and experienced an additional “visual capture” of the acupuncture needling, therefore exhibiting overall higher sympathetic activation to acupuncture stimulation. However, viewing a needle pricking a hand strengthens the perception of pain as well as the anticipation of forthcoming pain [34]. Acupuncture needles can intensify the fear of pain. Therefore, it is also possible that the autonomic response was not specific to acupuncture needling but a more common reaction to potentially pain-related cues. 

### 4.4. Clinical Implications of Bodily Awareness

Why would modifications to bodily self-awareness have important clinical implications? Recent research in the fields of neuroscience and neurology has shown that the conscious sense of one's physical self is closely linked to the physiological regulation of one's physical self [17, 27]. Moreover, aspects of “body awareness” have increasingly attracted the interest of researchers across many disciplines [3]. Disturbances in body awareness are thought to be related to a variety of diseases such as depression or schizophrenia, as well as to somatoform disorders and eating disorders [2]. Furthermore, individual differences in body awareness, which are closely related to symptom awareness, could possibly be one of the main factors contributing to different patient reactions to the same clinical treatment [4, 35], which has important consequences for the future of personalized medicine. 

Acupuncture is not just a treatment consisting of “simple needling.” Rather, it should be seen as a complex treatment comprising multimodal sensory stimulation interacting with various psychosocial factors [36]. According to this view, if acupuncture stimulation is applied, there is no single acupuncture effect but rather a total effect of acupuncture comprising different subsets of stimuli. These could include (a) bodily sensations including vision and somatosensation, as well as tactile sensation and the sensation of pain; (b) cognitive factors including attention, expectation, placebo effects, bodily self-awareness, and self-consciousness; and (c) sociocontextual factors such as perception of the clinical environment and the doctor-patient relationship. We suggest that distinguishing between the components of acupuncture effects in terms of bodily self-awareness could be a useful approach to understand the mechanisms of acupuncture treatment. 

## 5. Conclusion

Our focus in this study was to determine how responses to acupuncture needle stimulation would be specifically influenced by modifications of bodily self-awareness. Experiments involving illusions of body ownership have made it possible to manipulate different aspects of bodily self-awareness, revealing much about the physiological and neuroanatomical underpinnings of these illusions and explaining the multisensory mechanisms behind them [5, 6, 27, 37]. Our study is the first attempt to implement these experimental procedures to modify bodily self-awareness and to test psychophysiological responses to acupuncture needle stimulation. Relating and translating the knowledge of this field to acupuncture research could be valuable for a further understanding of underlying common mechanisms. Our findings suggest a new approach to scientific investigation of the effects and mechanisms of acupuncture.
